# Diagnostic and analytical performance of plasma pTau181 and 217 in a clinical laboratory

**DOI:** 10.1002/alz.086509

**Published:** 2025-01-09

**Authors:** Anna Mammel, Ging‐Yuek Robin Hsiung, Mary Encarnacion, Kelsey Hallett, Donald Biehl, Ali Mousavi, Hans Frykman

**Affiliations:** ^1^ Neurocode USA Inc., Bellingham, WA USA; ^2^ The University of British Columbia, Vancouver, BC Canada; ^3^ BC Neuroimmunology lab, Vancouver, BC Canada; ^4^ BC Neuroimmunology lab, vancouver, BC Canada; ^5^ Department of Medicine, University of British Columbia, Vancouver, Vancouver, BC Canada

## Abstract

**Background:**

Blood‐based biomarkers will be essential for providing clinicians an accessible and cost‐effective Alzheimer’s disease (AD) screening tool. Elevated levels of two phosphorylated tau biomarkers (pTau181 & pTau217) correlated with amyloid and tau‐PET consistent with AD diagnosis. We evaluated the analytical and clinical performance of each biomarkers using two different high‐sensitivity methodologies (CLEIA and Simoa®) in a single laboratory to compare the performance of pTau181 and 217 in a clinical (CLIA‐certified) laboratory.

**Method:**

Plasma pTau217 levels were measure using either the ALZpath pTau217 assay on the Quanterix HD‐X Simoa® platform or LUMIPULSE plasma pTau 217 on the Lumipulse G1200 platform. Plasma pTau181 levels were measured on the Simoa® platform using ADx Neurosciences antibodies.

Analytical performance including sensitivity, linearity, precision, accuracy, cross‐reactivity, interference, and sample stability was determined at Neurocode’s CLIA laboratory using CLIA guidelines.

Clinical testing was performed using a collection of amyloid PET confirmed samples (N = 260). And neuropathology confirmed samples collected from the University of British Columbia (UBC) at the time of initial diagnosis (N = 119).

**Result:**

pTau217 (AUC – 0.92 to 0.93) had enhanced clinical performance compared to pTau181 across platforms (AUC 0.81) and sample cohorts for diagnosing AD. pTau217 assays had a greater dynamic range (i.e. lower limit quantification) compared to pTau181, resulting in more substantial separation between lower‐limits and clinical decision points. pTau217 has a higher fold difference between PET positive and negative subjects regardless of methodology compared to pTau181. Sample stability was comparable across platforms and methodology. Between lot precision of pTau217 (≤ 13% CV) was improved compared to pTau181 (≤ 20% CV) on the Simoa platform.

**Conclusion:**

In conclusion, the superior clinical and analytical performance of pTau217 advocates for its use in clinical practice over pTau181. Plasma pTau217 is a robust screening tool that can be used by clinician for accurate and reliable Alzheimer's disease diagnosis.

